# Single Institution Experience with Immune Checkpoint Inhibitors in Vulvar and Vaginal Melanomas

**DOI:** 10.1155/2024/7327692

**Published:** 2024-08-13

**Authors:** Amrita Ladwa, Omar Elghawy, Varinder Kaur

**Affiliations:** ^1^ University of Virginia School of Medicine, Charlottesville, VA, USA; ^2^ Hospital of the University of Pennsylvania Internal Medicine Department, Philadelphia, PA, USA; ^3^ Department of Internal Medicine Division of Hematology and Oncology University of Virginia, Charlottesville, VA, USA

## Abstract

**Objectives:**

This study aimed to report clinical outcomes of patients with vaginal melanoma (VaM) or vulvar melanoma (VuM) who were treated with immune checkpoint inhibitors (ICI) and discuss the development of immune-related adverse events (irAE).

**Materials and Methods:**

This is a retrospective case series of patients diagnosed with VaM or VuM between July 2011 and September 2022 at the University of Virginia, Emily Couric Clinical Cancer Center. Patient demographics, disease characteristics, treatment outcomes, and adverse events were abstracted. The primary outcome was incidence of irAE.

**Results:**

Eight patients were included in this study, four with VaM and four with VuM. Most (*n* = 6) had local or regional disease at first presentation, and 25% (*n* = 2) presented with distant metastasis. All patients received a CTLA-4 inhibitor and 75% (*n* = 6) received PD-1 inhibitor alone or in combination with a CTLA-4 inhibitor. Most (75%, *n* = 6) patients experienced irAE. Of those who had irAE, 83% (*n* = 5) required therapy interruption or discontinuation. Most (66%, *n* = 4) underwent ICI rechallenge of which 75% (*n* = 3) experienced subsequent irAE. Of all patients in the series, 75% of patients (*n* = 6) had partial or complete response to ICI.

**Conclusion:**

This series is the first to detail incidence of irAEs and ICI rechallenges in vulvovaginal melanoma. Our findings indicate that while ICIs are effective, their use is associated with significant irAE development. Rechallenge of ICI after irAE is feasible but associated with risk of recurrent/new irAE. Further studies are needed to better quantify this risk.

## 1. Introduction

Vaginal melanoma (VaM) and vulvar melanoma (VuM) are rare malignancies that compose less than 1% of all melanomas [1–3]. These cancers are characterized by their aggressive nature and have poor prognoses [1, 2]. Five-year survival is dismal for these cancers (37–50% and 13–32% for VuM and VaM, respectively) [3]. The first-line treatment for VaM and VuM is surgical excision [2, 4–6], and systemic therapy for VaM and VuM has traditionally included IL-2, IFN-alpha, dacarbazine, temozolomide, and platinum/taxane regimens for recurrent or disseminated disease [1, 4, 7]. Radiotherapy has also been used for debulking synergistically with chemotherapy or immunotherapy (IO) [7, 8]. However, given the poor outcomes with these standard treatments, palliative care has remained a pillar of treatment.

Immune checkpoint inhibitors (ICI) have shown promise in multiple cancers and are increasingly used in progressive or metastatic disease [9, 10]. ICI has shown dramatic improvements in overall survival (OS) in cutaneous melanoma [4] and modest improvements in OS for mucosal melanoma [10, 11]. Vulvovaginal melanoma is rarely isolated in large studies, but one prospective report by Albert et al. found a higher 2-year overall survival in metastatic disease in patients who were treated with versus without ICI (33% vs. 12%), although this difference was not statistically significant [12].

While ICI may hold promise for VaM and VuM, this therapy is not without risk. Immune-related adverse events (irAE) are toxicities due to ICI therapy that can affect any organ system. They are difficult to predict, range in severity, and may require treatment discontinuation and/or prolonged steroids. In patients with a history of irAE, the decision to rechallenge with ICI must consider the risk of new or worsening irAE. Studies of ICI rechallenge in other cancers have found potential survival benefit but with mixed results on the incidence and severity of subsequent irAEs [13, 14]. No studies discuss rechallenge in vulvovaginal melanoma and no guidance is available to determine which patients would benefit from rechallenge. In this retrospective study, we describe eight cases of VaM and VuM treated with ICI and discuss incidence of irAE and outcomes with ICI rechallenge.

## 2. Methods

A single center, retrospective observational study was performed by searching the electronic medical record for all patients that received CTLA-4 inhibitors or PD-1 inhibitors between July 2011 and September 2022 at the University of Virginia, Emily Couric Clinical Cancer Center. Data abstraction was conducted manually to collect patient information including demographics, cancer diagnosis, staging information, comorbidities, treatments administered and subsequent toxicities, and re-challenge with subsequent treatments. Staging was determined by the American Joint Committee on Cancer (AJCC) system, version 8 [15]. Toxicity from ICI was graded by Common Terminology Criteria for Adverse Events, version 5 [16]. Statistical analysis was performed using XLSTAT 2024 (Addinsoft, Paris, France). The study was approved by the University of Virginia Institutional Review Board.

## 3. Results

### 3.1. Demographics and Disease Characteristics

Of the 1979 patients treated with ICI at our institution between 2011 and 2023, eight were diagnosed with VaM (*n* = 4) or VuM (*n* = 4). Demographic and disease information is listed in Table 1. At diagnosis, the median age was 67 years (range, 31–61 years) and median BMI was 28.9 (range, 20.85–41.97). Seven patients were white, and one patient was African American. Most patients had no history of autoimmune disease; patient 2 had a history of Raynaud's disease.

At diagnosis of VaM/VuM, most patients presented with new-onset symptoms (*n* = 7) including weight loss (*n* = 2), abdominal pain (*n* = 1), vaginal pruritus (*n* = 1), new vulvar pigmentation (*n* = 2), and mass with (*n* = 2) or without (*n* = 1) ulceration. One patient had a pigmented lesion identified on routine gynecologic examination. Histologic subtypes were varied and included mucosal (*n* = 3), superficial spreading (*n* = 2), lentiginous (*n* = 1), nodular (*n* = 1), and unspecified (*n* = 1) melanoma. Half of the patients (*n* = 4) presented with AJCC stage III or higher at diagnosis; of those, two presented with stage IV disease due to nodal and/or hepatic metastasis. PD-L1 testing was performed on one patient with TPS <1. All patients (*n* = 8) were wild-type for BRAF. Most patients (*n* = 7) developed distant metastasis with a median time to metastasis of 6 months (range, 0–41 months).

### 3.2. Treatment

Most patients (*n* = 6) underwent surgical excision of the primary site. Surgical interventions included wide local excision alone (*n* = 2) or excision/vulvectomy/vaginectomy with nodal dissection (*n* = 4). Patient 1 did not have surgery due to the large size of her tumor (7.2 × 7.3 × 4.4 cm) and presence of retroperitoneal lymph node metastasis. Patient 3 was offered pelvic exenteration or up-front systemic therapy, and she chose to proceed with systemic therapy. No patients underwent pelvic exenteration. Details of treatments and outcomes are listed in Table 2.

Most patients (*n* = 6) had stage IV disease at ICI initiation. ICI was utilized as first-line therapy for three patients, second-line therapy for three patients, and third-line therapy for two patients. All patients received CTLA-4 inhibitors, and six received PD-1 inhibitors alone or in combination with CTLA-4 inhibitors. Other first-line treatments included high-dose IL-2 (patient 1), interferon (patient 2), imatinib (patients 6 and 7), and a melanoma vaccine as part of a clinical trial (patient 8).

### 3.3. Outcomes

Six patients experienced partial or complete response to ICI. Patients 3, 4, and 5 received first-line ipilimumab/nivolumab and demonstrated sustained pathologic complete response with no progression at median follow-up of 77 months. Patients 2 and 8 received third-line ipilimumab with or without nivolumab and had progressive disease.

### 3.4. Immune-Related Adverse Events

Details about irAE are listed in Table 3. Six patients experienced irAE, all while on ipilimumab ± nivolumab. irAE led to therapy interruption in four cases with permanent discontinuation in one case (patient 2). No irAE-related deaths were observed. Median time to irAE after ICI treatment initiation was 23.5 days (range, 14–91 days). Four patients underwent ICI rechallenge (patients 3, 4, 7, and 8), of which three experienced new irAEs (patients 3, 4, and 8).

Patient 2 developed a grade 3 rash following one cycle of ipilimumab. ICI was stopped, she was treated with prednisone, and the rash resolved over the following two months. The patient passed away one month later from unknown causes.

Patient 3 developed grade 4 transaminitis and diabetic ketoacidosis from insulin-dependent diabetes after two cycles of ipilimumab/nivolumab. She was started on insulin, treated with steroids, and ICI was held for two months. Transaminitis responded well to a course of steroids, and she remained on a stable insulin regimen. Two months after the development of these irAE, she was rechallenged with nivolumab and transitioned to nivolumab/relatlimab for progression. Nivolumab/relatlimab was discontinued after 12 months due to progression, and two months after this she developed grade 2 transaminitis. This irAE was thought to be a residual effect of ICI and she was treated with steroids and mycophenolate mofetil. She tolerated this treatment well and had slow resolution of transaminitis.

Patient 4 developed thrombocytopenia and grade 1 transaminitis after three cycles of ipilimumab/nivolumab. She was hospitalized, received high-dose steroids, cryoprecipitate, and IVIG. The irAE eventually resolved, and ICI was held for nine months until she experienced disease progression. She underwent ICI rechallenge with nivolumab and developed grade 2 colitis and uveitis after one cycle. She was treated with topical steroids for uveitis and oral steroids for colitis, and ICI therapy was continued. The uveitis improved transiently but she eventually developed cataracts necessitating surgical correction. She had waxing and waning colitis that only improved with prolonged steroids and mycophenolate mofetil. Over the next four months, she had multiple ICI pauses for cumulative toxicity and ultimately decided to discontinue treatment.

Patient 7 developed vitiligo and hypothyroidism after seven cycles of single-agent nivolumab. ICI was held for a month while she initiated levothyroxine and rechallenge with nivolumab was uncomplicated. She continued to require thyroid replacement until her passing one year later.

Patient 8 developed actinic keratoses following one cycle of ipilimumab/nivolumab. She was treated with steroids and cryotherapy, and ICI was continued. After her third cycle of ipilimumab/nivolumab, she developed grade 3 colitis. Treatment was held, she received steroids, and the colitis resolved appropriately. Four months later she restarted nivolumab monotherapy. After one dose, she developed grade 3 actinic keratoses. ICI was held, she was treated with oral steroids, and had partial improvement. Biopsy of the lesions returned as squamous cell carcinoma. She declined surgery, so the lesions were treated with acitretin. This therapy was tolerated well, and the lesions appeared to resolve clinically. Due to progression of melanoma, she initiated pembrolizumab and had no further irAE. She eventually developed new metastases and passed away.

## 4. Discussion

In this series, we describe eight cases of vulvovaginal melanoma treated with ICI and explore incidence of irAE before and after ICI rechallenge. ICI resulted in treatment response in 75% of patients but was complicated by irAE in 67% of that subset. This incidence is similar to reported values for cutaneous melanoma [17, 18]. Most patients who experienced irAE had ≥grade 3 reactions (67%) and/or required therapy interruption or discontinuation (83%), and half had incomplete resolution with sequelae (actinic keratoses transformed to squamous cell cancer) or nonresolution (insulin-dependent diabetes, hypothyroidism) of the irAE (50%). Of those who experienced irAE, most were rechallenged with ICI (67%) and developed subsequent irAEs (75%). Compared to two similar case series of vulvovaginal melanoma, combination ICI was used more frequently (50% vs 0–9%) and incidence of severe irAE was higher (50% vs 0–13%) [1, 4]. However, studies on irAE in vulvovaginal melanoma are very limited and no other studies to the authors' knowledge provide information regarding ICI rechallenge.

Literature about irAEs in other neoplasms seems to suggest a positive prognostic association with irAE [17, 18]. For example, one paper found that patients with irAE had significantly higher median OS compared to those who did not experience irAE (56.3 vs 18.5 months) [17]. Outcomes regarding OS cannot be drawn in our cohort due to small sample size and inconsistent follow-up period. Regardless, ICI carries a risk of significant irAE that may require therapy interruption, prolonged steroids, and discontinuation of treatment. In severe cases, irAE may be permanent (e.g., insulin-dependent diabetes) or lead to significant morbidity and mortality. Rechallenge of ICI following irAE appears possible in a proportion of patients with vulvovaginal melanoma and warrants careful clinical evaluation on an individual basis.

This is one of few series to report on vulvovaginal melanoma treated by ICI, and it is the first to discuss irAE incidence during ICI rechallenge. Our findings show that rechallenge of ICI confers risk of new and/or high-grade irAE. This study is limited due to its small sample size and heterogeneity within the study cohort in terms of disease characteristics and prior treatment. Furthermore, due to its retrospective design, a lack of standardized intervals for disease assessment was utilized. The retrospective design of this study also limits capturing specific details regarding adverse events and tolerability within the study cohort. Further research on a larger scale is indicated to stratify this risk by patient and disease characteristics. Our study was limited in scope as a small, single institution retrospective series with variable follow-up duration. Cycles and dosing of ICI were not reported or considered during analysis.

## 5. Conclusion

Here, we report on eight patients with vulvovaginal melanoma at a single institution treated with ICI. Our findings emphasize the need for study on the risks and possible benefits of ICI rechallenge in patients with a history of irAE.

## Figures and Tables

**Table 1:** Patient demographics and disease characteristics.

Patient	Age	ECOG	Race	Ethnicity	Melanoma subtype	Site	Breslow depth	AJCC stage at diagnosis	AJCC stage at ICI initiation	Developed metastasis?	Time to metastasis (months)	PD-L1	C-KIT	NRAS	BRAF
1	69	2	African American	Non-Hispanic	Unknown	Vaginal	Unknown	IV	IV	Y	0	NA	UNK	UNK	WT
2	53	1	White	Non-Hispanic	Mucosal	Vaginal	1.5 mm	IB	IV	Y	28	NA	WT	UNK	WT
3	69	0	White	Non-Hispanic	Mucosal	Vaginal	2.1 mm	IIB	IIB	N	NA	NA	K642E mutation	UNK	WT
4	31	0	White	Non-Hispanic	Mucosal	Vaginal	43 mm	IV	IV	Y	0	NA	UNK	G12D missense	WT
5	70	0	White	Non-Hispanic	Nodular	Vulvar	1.95 mm	IIIA	IIIA	Y	9	Negative	UNK	WT	WT
6	54	0	White	Non-Hispanic	Superficial spreading	Vulvar	4.9 mm	IIIB	IV	Y	3	NA	Exon 11 mutation	UNK	WT
7	71	0	White	Non-Hispanic	Superficial spreading	Vulvar	0.54 mm	IA	IV	Y	30	NA	L576P Exon 11 mutation	UNK	WT
8	65	0	White	Non-Hispanic	Lentiginous	Vulvar	2.0 mm	IIA	IV	Y	41	NA	UNK	UNK	WT

ECOG: Eastern Cooperative Oncology Group.

**Table 2:** Treatments received and patient outcomes.

Patient	Initial surgery	Radiotherapy site	ICI line	Treatment prior to ICI	All ICI	irAE	Best response on ICI	Status
1	None	Pelvis Inguinal lymph nodes	2	IL-2	Ipilimumab	N	PR	Deceased
2	R1	Vagina	3	IFN, IL-2	Ipilimumab	Y	PD	Deceased
3	None	Pelvis Inguinal lymph nodes	1	N/A	Ipilimumab Nivolumab	Y	CR	Alive, in treatment
4	R2	None	1	N/A	Ipilimumab Nivolumab	Y	CR	Alive, in remission
5	R0	Inguinal lymph nodes	1	N/A	Ipilimumab Nivolumab	Y	CR	Alive, in remission
6	R0	Brain	2	Imatinib	Ipilimumab Pembrolizumab	N	PR	Deceased
7	R0	Brain	2	Imatinib	Ipilimumab Nivolumab	Y	PR	Deceased
8	R0	None	3	LPV and TLR agonists, IL-2	Ipilimumab Nivolumab Pembrolizumab	Y	PD	Deceased

**Table 3:** Details of immune-related adverse events (irAE).

Patient	ICI causing irAE	irAE	Grade	Treatment held	irAE management	irAE resolved	ICI rechallenge	ICI resumed	Subsequent irAE	Subsequent therapy
1	N/A	None	N/A	N/A	N/A	N/A	N/A	N/A	N/A	N/A
2	Ipilimumab	Dermatitis	Grade 3	Y	Steroids	Y	N	N/A	N/A	N/A
3	Ipilimumab Nivolumab	Diabetes Transaminitis	No established criteria Grade 4	Y	Insulin and steroids	N	Y	Nivolumab	Grade 2 transaminitis	Nivolumab and relatlimab
4	Ipilimumab Nivolumab	Thrombocytopenia Transaminitis	No established criteria Grade 1	Y	Steroids	Y	Y	Nivolumab	Grade 2 uveitis Grade 2 colitis	N/A
5	Ipilimumab Nivolumab	Dermatitis	Grade 3	N	Steroids	Y	N/A	N/A	N/A	N/A
6	N/A	None	N/A	N/A	N/A	N/A	N/A	N/A	N/A	N/A
7	Nivolumab	Hypothyroidism Vitiligo	Grade 2 Grade 1	Y	Levothyroxine	N	Y	Nivolumab	N	Ipilimumab
8	Ipilimumab Nivolumab	Keratoses Colitis	Grade 2 Grade 1	Y	Cryotherapy Steroids	Y	Y	Ipilimumab Nivolumab	Squamous cell carcinoma	Pembrolizumab

## Data Availability

The data that support the findings of this study are available from the corresponding author upon reasonable request.
